# Casual associations between frailty and nine mental disorders: bidirectional Mendelian randomisation study

**DOI:** 10.1192/bjo.2024.835

**Published:** 2025-02-03

**Authors:** Yong Zhou, Jiayue Duan, Jiayi Zhu, Yunying Huang, Tao Tu, Keke Wu, Qiuzhen Lin, Yingxu Ma, Qiming Liu

**Affiliations:** Department of Cardiovascular Medicine, The Second Xiangya Hospital of Central South University, Changsha, China; Department of Endocrinology, Key Laboratory of Endocrinology, Ministry of Health Peking Union Medical College Hospital, Chinese Academy of Medical Sciences & Peking Union Medical College, Beijing, China

**Keywords:** Depressive disorders, genetics, genomics, suicide, psychological disorders

## Abstract

**Background:**

An increasing number of observational studies have reported associations between frailty and mental disorders, but the causality remains ambiguous.

**Aims:**

To assess the bidirectional causal relationship between frailty and nine mental disorders.

**Method:**

We conducted a bidirectional two-sample Mendelian randomisation on genome-wide association study summary data, to investigate causality between frailty and nine mental disorders. Causal effects were primarily estimated using inverse variance weighted method. Several secondary analyses were applied to verify the results. Cochran's *Q*-test and Mendelian randomisation Egger intercept were applied to evaluate heterogeneity and pleiotropy.

**Results:**

Genetically determined frailty was significantly associated with increased risk of major depressive disorder (MDD) (odds ratio 1.86, 95% CI 1.36–2.53, *P* = 8.1 × 10^−5^), anxiety (odds ratio 2.76, 95% CI 1.56–4.90, *P* = 5.0 × 10^−4^), post-traumatic stress disorder (PTSD) (odds ratio 2.56, 95% CI 1.69–3.87, *P* = 9.9 × 10^−6^), neuroticism (*β* = 0.25, 95% CI 0.11–0.38, *P* = 3.3 × 10^−4^) and insomnia (*β* = 0.50, 95% CI 0.25–0.75, *P* = 1.1 × 10^−4^). Conversely, genetic liability to MDD, neuroticism, insomnia and suicide attempt significantly increased risk of frailty (MDD: *β* = 0.071, 95% CI 0.033–0.110, *P* = 2.8 × 10^−4^; neuroticism: *β* = 0.269, 95% CI 0.173–0.365, *P* = 3.4 × 10^−8^; insomnia: *β* = 0.160, 95% CI 0.141–0.179, *P* = 3.2 × 10^−61^; suicide attempt: *β* = 0.056, 95% CI 0.029–0.084, *P* = 3.4 × 10^−5^). There was a suggestive detrimental association of frailty on suicide attempt and an inverse relationship of subjective well-being on frailty.

**Conclusions:**

Our findings show bidirectional causal associations between frailty and MDD, insomnia and neuroticism. Additionally, higher frailty levels are associated with anxiety and PTSD, and suicide attempts are correlated with increased frailty. Understanding these associations is crucial for the effective management of frailty and improvement of mental disorders.

Frailty is a prevalent geriatric syndrome, which is characterised by diminished physiological reserves across multiple systems, rendering individuals less resilient to stressors and increasing their vulnerability to adverse health outcomes.^1^ Globally, 18.7–53.1% of community-dwelling older adults are severely affected by pre-frailty, with frailty being observed in 4.2–59.1% of cases.^2^ Frailty is notably associated with an elevated risk of mortality and poses an escalating global health challenge.^1^ Investigating the potential relationship between frailty and frailty-related diseases could provide valuable insights for tailoring individualised management and implementing early interventions for this population.

Mental disorders, widely considered as a significant public health concern, have risen to prominence as one of the primary contributors to global disability.^3^ It is estimated that more than 25% of the population experience psychiatric disorders, constituting 19% of people living with a disability.^4^ Emerging evidence from epidemiological studies indicates a robust association between frailty and mental disorders.^5–8^ A multicentre, cross-sectional study involving individuals admitted to hospital with COVID-19 demonstrated that those living with frailty exhibited a significantly increased likelihood of experiencing symptoms of anxiety, depression and post-traumatic stress disorder (PTSD),^8^ which showed correlation between frailty and mental disorders. A longitudinal cohort study involving 5303 older Chinese adults found that individuals living with pre-frailty and frailty were at higher risk of developing depressive symptoms compared with their more robust counterparts,^7^ which suggested potential causal relationships between frailty and mental disorders. Besides, an analysis of a national cohort consisting of 2 858 876 participants suggested that people with pre-frailty to severe frailty were at a heightened risk of suicide attempt.^5^ Additionally, frailty is progressively recognised as a valuable clinical metric within psychiatric healthcare. A recent systematic review encompassing 25 studies highlighted that the prevalence of frailty in individuals with severe mental illness ranged from 10.2 to 89.7%.^9^ A longitudinal study recording 297 380 individuals over 12.19 years observed elevated levels of frailty among those with depression, bipolar disorder or anxiety disorders.^6^ Furthermore, genome-wide association studies (GWASs) of frailty have shed light on the role of mental health and underscored pathways linked to brain function in ageing.^10^ However, the causal relationship between frailty and mental disorders remains uncertain, as existing evidence from observational studies cannot fully account for reverse causality and confounding factors. Substantial uncertainty persists regarding the existence of a bidirectional causal association or whether coexistence is attributable to confounding or shared risk factors, such as obesity and smoking.

Mendelian randomisation is a powerful genetic epidemiology method employed to ascertain causal relationships between exposures and their corresponding outcomes.^11^ It operates by utilising genetic variations, typically single nucleotide polymorphisms (SNPs), as instrumental variables. Importantly, Mendelian randomisation capitalises on the principle that genetic variants are equally, randomly and independently distributed at conception, thereby effectively mitigating the influence of confounding factors and the possibility of reverse causality.^11^ Mendelian randomisation has been widely used to investigate causal relationships between frailty and various diseases, including mental disorders.^10,12^ Although previous Mendelian randomisation studies have primarily focused on depression, anxiety, bipolar disorder and schizophrenia, we extended this analysis to include neuroticism, subjective well-being, PTSD, insomnia and suicide attempt. Our goal was to provide a more comprehensive understanding of the association between frailty and mental health. In this study, we conducted a bidirectional Mendelian randomisation analysis, leveraging the latest GWASs, to comprehensively elucidate the potential causal relationship between genetically determined frailty and nine mental disorders.

## Method

### Study design

**Fig. 1:**
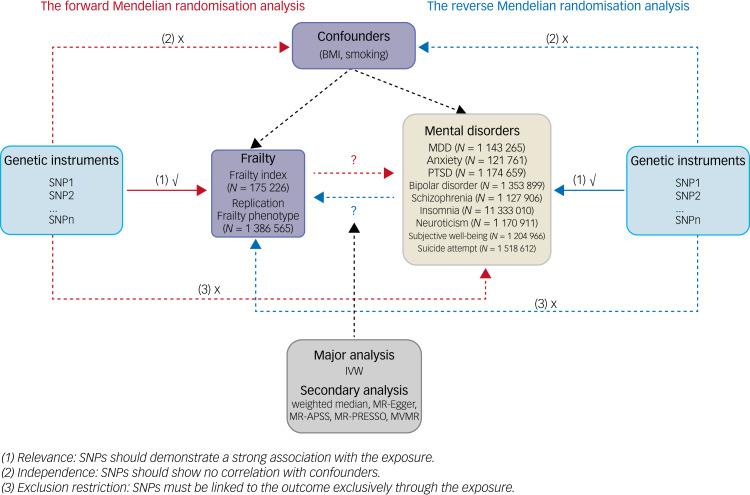
Overview of this Mendelian randomisation study design. BMI, body mass index; MDD, major depressive disorder; IVW, inverse-variance weighted; MR-APSS, Mendelian randomisation for causal inference accounting for pleiotropy and sample structure; MR-Egger, Mendelian randomisation Egger; MR-PRESSO, Mendelian randomisation pleiotropy residual sum and outlier; MVMR, multivariable Mendelian randomisation; PTSD, post-traumatic stress disorder; SNP, single nucleotide polymorphism.

This study employed a bidirectional two-sample Mendelian randomisation design, outlined in Fig. 1. SNPs served as instrumental variables in this Mendelian randomisation analysis. To ensure the validity of causal inferences drawn from Mendelian randomisation analyses, the instrumental variables must satisfy three fundamental assumptions: (a) the relevance assumption, implying that SNPs should exhibit a robust association with the exposure phenotype; (b) the independence assumption, indicating that instrumental variables should not be correlated with confounding factors; and (c) the exclusion restriction assumption, positing that the causal pathway should operate solely through the exposure of interest.^11^ The Mendelian randomisation analyses were conducted in two directions: (a) with frailty as the exposure to assess whether individuals with higher frailty were more susceptible to mental disorders, and (b) with frailty as the outcome to evaluate whether individuals with mental disorders were more frail. Our study adhered to the reporting guidelines outlined in the Strengthening the Reporting of Observational Studies in Epidemiology using Mendelian randomisation statement.

### Data sources

The data-sets utilised in this study were sourced from publicly available repositories, and ethical approvals were gained in all original studies.

Frailty is commonly defined using two primary instruments: the frailty index or the frailty phenotype.^10^ For primary analyses, data-sets of frailty assessed by frailty index were applied. Summary statistics for the frailty index were acquired from the latest GWAS meta-analysis, encompassing 175 226 individuals of European descent from the UK Biobank and Swedish TwinGene.^10^ The frailty index was computed based on the accumulation of 44–49 self-reported health deficits experienced throughout one's life course. For replication analyses, we used another GWAS of frailty measured by frailty phenotype. The frailty phenotype was assessed using five criteria: weight loss, exhaustion, low physical activity, slow walking speed and weak grip strength. Summary data for the frailty phenotype were obtained from a recent large-scale GWAS, encompassing 386 565 participants of European descent registered in the UK Biobank.^13^

Summary data for mental disorders, including major depressive disorder (MDD),^14^ anxiety,^15^ PTSD,^16^ bipolar disorder^17^ and schizophrenia,^18^ were sourced from the Psychiatric Genomics Consortium. Because of the limited number of participants for summary data on anxiety from the Psychiatric Genomics Consortium, we further applied summary statistics from the FinnGen consortium to verify the results. Non-gender-specific summary statistics of European ancestry were selected, and no sample overlap was identified between these data-sets and those related to frailty studies. Additionally, summary statistics for insomnia symptoms were acquired from a recent GWAS meta-analysis conducted by Jansen et al, involving a substantial cohort of 1 331 010 individuals.^19^ We retrieved summary data for subjective well-being and neuroticism from the Social Science Genetics Association Consortium.^20^ For suicide attempt, GWAS summary statistics were obtained from the International Suicide Genetics Consortium data repository, which represents the largest GWAS effort focused on suicide attempt.^21^ Further details are presented in Table 1.

**Table 1:** Information on GWAS summary data in the Mendelian randomisation study

Phenotype	Consortium	Sample size (overall or case/control)	Population	Year	PMID
Frailty index	UK Biobank and Swedish TwinGene	175 226	European	2021	34431594
Frailty phenotype	UK Biobank	386 565	European	2023	36928559
MDD	PGC	45 591/97 674	European	2018	29700475
Anxiety	PGC	7016/14 745	European	2016	26754954
Anxiety	FinnGen consortium	31 780/403 817	European	2023	36653562
PTSD	PGC	23 212/151 447	European	2019	31594949
Bipolar disorder	PGC	40 463/313 436	European	2021	34002096
Schizophrenia	PGC	52 017/75 889	European	2022	35396580
Neuroticism	SSGAC	170 911	European	2016	27089181
Subjective well-being	SSGAC	204 966	European	2016	27089181
Insomnia	UK Biobank and 23andme	1 331 010	European	2019	30804565
Suicide attempt	ISGC	26 590/492 022	European	2022	34861974

GWAS, genome-wide association study; PMID, PubMed ID number; MDD, major depressive disorder; PGC, Psychiatric Genomics Consortium; PTSD, post-traumatic stress; SSGAC, Science Genetics Association Consortium; ISGC, International Suicide Genetics Consortium.

### Selection of the genetic instruments

To identify appropriate genetic instruments, we commenced by pinpointing genome-wide significant SNPs (*P* < 5 × 10^−^⁸) that displayed strong associations with each of the studied phenotypes. However, to encompass a wider spectrum of SNPs potentially associated with mental disorders, we adopted more lenient thresholds: *P* < 5 × 10^−^⁵ for anxiety; *P* < 5 × 10^−^⁶ for PTSD, suicide attempt and subjective well-being; and *P* < 5 × 10^−^⁷ for MDD and neuroticism. For insomnia, we used SNPs associated with insomnia identified by Jansen and colleagues.^19^ It is worth noting that relaxing the threshold for genetic instruments is a recognised practice in psychiatric Mendelian randomisation studies when there is a scarcity of significant SNPs.^22^ Subsequently, we calculated the linkage disequilibrium among the selected SNPs, utilising the linkage disequilibrium metric (*r*²) to distinguish independent SNPs (linkage disequilibrium *r*² < 0.001 within a 10 000 kb window) and eliminate dependent ones. The PLINK clumping method was employed for this purpose. To mitigate bias resulting from weak instrumental variables, we determined the *F*-statistic for each SNP individually, excluding instruments with an *F*-statistic <10. The *R*², representing the variance explained by each genetic instrument, was estimated using the formula: 2 × EAF × (1–EAF) × *β*². The *F*-statistic was computed as *F* = *R*² × (*N*–2)/(1–*R*²) to gauge the strength of the genetic instrument. Here, EAF signified the effect allele frequency, *β* denoted the effect size of instrumental variables and *N* represented the sample size of the GWAS. Following the harmonisation of exposure and outcome data-sets, we removed palindromic and weak instrumental variants, retaining the remaining SNPs for Mendelian randomisation analyses. Additionally, we examined whether the selected SNPs were associated with obesity or smoking, by using Phenoscanner (details given below; http://www.phenoscanner.medschl.cam.ac.uk/), a tool that evaluates selected genetic instruments and their proxies (*r*² > 0.8) for associations with secondary phenotypes (*P* < 5 × 10^−^⁸).

### Mendelian randomisation analysis

For the primary analysis, we employed the random-effects inverse-variance weighted (IVW) method to derive Mendelian randomisation estimates. This method combines the Wald ratio estimates of the causal effects of each SNP, assuming the validity of all SNPs. To assess the robustness of the Mendelian randomisation results, we also utilised several secondary methods: the weighted median, Mendelian randomisation Egger (MR-Egger) and Mendelian randomisation pleiotropy residual sum and outlier (MR-PRESSO) approaches. These secondary methods offered more reliable estimates in a broader range of situations, albeit with slightly lower efficiency (resulting in wider confidence intervals). The weighted median method can produce valid Mendelian randomisation estimates even in the presence of horizontal pleiotropy, when up to 50% of the included instruments are invalid. MR-Egger regression can provide valid Mendelian randomisation estimates even when horizontal pleiotropy is present, as long as the pleiotropic effects of the SNPs are independent of their genetic associations with the exposure. The MR-PRESSO approach can identify outliers and provide a causal estimate free from the influence of these outliers.

In this study, we recognised smoking and obesity as significant confounding factors in the relationship between frailty and mental disorders. To address this, we conducted an analysis in which we additionally excluded SNPs demonstrating genome-wide significant associations with smoking and obesity-related traits, as revealed by Phenoscanner results. Moreover, we applied multivariable Mendelian randomisation (MVMR), an extension of Mendelian randomisation that employs genetic variants linked to multiple, conceivably interrelated exposures. This approach enabled us to discern the joint causal effects of multiple risk factors. As a result, we performed MVMR analyses to account for potential pleiotropy arising from smoking and obesity. The summary statistics for these potential confounding factors were sourced from the IEU OpenGWAS project (https://gwas.mrcieu.ac.uk/) with the respective GWAS identifiers ieu-b-40 and ieu-b-4877. The new estimates reflected the direct causal effect while keeping smoking and obesity constant.

### Pleiotropy and sensitivity analysis

The intercept in MR-Egger regression serves as an indicator of the average pleiotropic effect across the instrumental variables. Hence, if the MR-Egger test yields an intercept significantly different from zero, this signals the presence of pleiotropy. To gauge heterogeneity within the Mendelian randomisation analysis, we employed Cochran's *Q*-statistic. We conducted a leave-one-out analysis to scrutinize whether the overall estimates were unduly influenced by a single SNP. As some bidirectional associations were observed, we further used the Steiger filtering, which removed SNPs explaining more variance of the outcome than the exposure to ensure the correct direction of inferred causal associations. To address the potential influence of sample overlap on the estimates, we utilised a recently developed method, Mendelian randomisation for causal inference accounting for pleiotropy and sample structure (MR-APSS).^23^ MR-APSS employed a foreground-background model to disentangle observed SNP sizes. The background model addresses latent confounding factors in GWAS summary statistics, including correlated pleiotropy and sample structure, such as potential sample overlap. Adhering to the assumptions of linkage disequilibrium score regression, the background model incorporates pleiotropy and sample structure through genome-wide summary statistics. This method explicitly considered sample overlap and was applied to recompute the Mendelian randomisation estimates by using default parameters.^23^

All statistical analyses were conducted with R version 4.3.2 (see https://www.r-project.org/) and specific packages, including TwosampleMR version 0.5.7 for Windows (see https://mrcieu.github.io/TwoSampleMR/), MendelianRandomization version 0.8.0 for Windows (see https://CRAN.R-project.org/package=MendelianRandomization) and MR-PRESSO version 1.0 for Windows (see https://github.com/rondolab/MR-PRESSO). Results were presented as *β* when the outcome was continuous or ordinal, and as odds ratios for dichotomous outcomes. Statistical power for Mendelian randomisation analyses was calculated with an online tool (https://sb452.shinyapps.io/power/). To account for multiple testing, we applied a conservative Bonferroni-corrected threshold (*P* < 0.0028), using the IVW method in our primary analysis. This stringent threshold was adopted because we investigated the 18 associations between frailty and nine mental disorders in both causal directions. *P*-values ranging from 0.0028 to 0.05 were considered as suggestive relationships.

## Results

The number of SNPs used as genetic instruments varied, ranging from 11 (MDD) to 149 (insomnia). Detailed lists of these SNPs are provided in Supplementary Table 1 available at https://doi.org/10.1192/bjo.2024.835. All *F*-statistics associated with these SNPs exceeded 10, indicating the absence of weak instrument bias. Most of the associations examined exhibit robust statistical power, with over 80% power in the primary data-sets, as outlined in Supplementary Table 2.

### Causal associations of frailty on mental disorders

Using the IVW method, genetically predicted frailty index exhibited significant associations with an increased risk of MDD (odds ratio 1.86, 95% CI 1.36–2.53, *P* = 8.1 × 10^−5^), anxiety (odds ratio 2.76, 95% CI 1.56–4.90, *P* = 5.0 × 10^−4^), PTSD (odds ratio 2.56, 95% CI 1.69–3.87, *P* = 9.9 × 10^−6^), neuroticism (*β* = 0.25, 95% CI 0.11–0.38, *P* = 3.3 × 10^−4^) and insomnia (*β* = 0.50, 95% CI 0.25–0.75, *P* = 1.1 × 10^−4^), as presented in Table 2. Additionally, we observed a suggestive association of genetically proxied frailty with a higher risk of suicide attempt (odds ratio 1.58, 95% CI 1.05–2.36, *P* = 0.027). These findings retained their significance when applying pleiotropy-robust methods, including the weighted median and MR-PRESSO (both raw and outlier-corrected), except for MR-Egger, which exhibited lower precision compared with the other methods. For anxiety, comparable findings were obtained from the summary statistics of the FinnGen consortium, as detailed in Supplementary Table 3. Besides, similar results were obtained when the obesity- and smoking-related SNPs were excluded (Supplementary Table 4). Furthermore, the results from the MVMR analyses adjusting for body mass index or/and smoking, remained consistent with the primary IVW results (Supplementary Table 5). Notably, no statistically significant causal associations were identified between frailty and bipolar disorder, schizophrenia and subjective well-being.

**Table 2:** Mendelian randomisation estimates for the causal associations of frailty on mental disorders

Exposure	Outcome	Method	*β* (95% CI)^a^	Odds ratio (95% CI)^b^	*P*-value
Frailty index	MDD	IVW	−	1.86 (1.36–2.53)	8.1 × 10^5^
		Weighted median	−	1.74 (1.20–2.52)	3.4 × 10^3^
		MR-Egger	−	0.97 (0.03–27.17)	0.99
		MR-PRESSO	−	1.86 (1.36–2.53)	1.9 × 10^−3^
	Anxiety	IVW	−	2.76 (1.56–4.90)	5.0 × 10^−4^
		Weighted median	−	3.27 (1.18–9.08)	0.023
		MR-Egger	−	0.36 (0.00–1566.32)	0.82
		MR-PRESSO	−	2.76 (1.56–4.90)	4.6 × 10^−3^
	PTSD	IVW	−	2.56 (1.69–3.87)	9.9 × 10^−6^
		Weighted median	−	2.09 (1.19–3.66)	0.010
		MR-Egger	−	1.09 (0.17–7.07)	0.93
		MR-PRESSO	−	2.56 (1.69–3.87)	6.9 × 10^−4^
	Bipolar disorder	IVW	−	1.31 (0.80–2.13)	0.29
		Weighted median	−	1.24 (0.80–1.98)	0.36
		MR-Egger	−	0.61 (0.05–7.40)	0.71
		MR-PRESSO^c^	−	1.46 (0.97–2.21)	0.10
	Schizophrenia	IVW	−	1.45 (0.83–2.54)	0.20
		Weighted median	−	1.19 (0.75–1.87)	0.63
		MR-Egger	−	15.17 (1.52–151.89)	0.043
		MR-PRESSO^c^	−	1.21 (0.78–1.88)	0.42
	Neuroticism	IVW	0.25 (0.11–0.38)	−	3.3 × 10^−4^
		Weighted median	0.24 (0.09–0.40)	−	1.9 × 10^−3^
		MR-Egger	−0.50 (−1.84 to 0.85)	−	0.48
		MR-PRESSO	0.25 (0.11–0.38)	−	3.7 × 10^−3^
	Subjective well-being	IVW	−0.18 (−0.38 to 0.03)	−	0.087
		Weighted median	−0.11 (−0.28 to 0.07)	−	0.24
		MR-Egger	2.11 (−0.02 to 4.24)	−	0.12
		MR-PRESSO^c^	−0.18 (−0.38 to 0.03)	−	0.15
	Insomnia	IVW	0.50 (0.25–0.75)	−	1.1 × 10^−4^
		Weighted median	0.25 (0.03–0.47)	−	0.027
		MR-Egger	−0.54 (−1.53 to 0.44)	−	0.30
		MR-PRESSO^c^	0.54 (0.28–0.80)	−	1.8 × 10^−3^
	Suicide attempt	IVW	−	1.58 (1.05–2.36)	0.027
		Weighted median	−	1.67 (1.07–2.59)	0.023
		MR-Egger	−	24.32 (0.44–1353.11)	0.15
		MR-PRESSO	−	1.58 (1.05–2.36)	0.047

MDD, major depressive disorder; IVW, inverse-variance weighted; MR-Egger, Mendelian randomisation Egger; MR-PRESSO, Mendelian randomisation pleiotropy residual sum and outlier; PTSD, post-traumatic stress disorder.

a. *β* is presented for the analyses of continuous/ordinal outcomes.

b. Odds ratio is presented for the analyses of binary/dichotomous outcomes.

c. MP-PRESSO outlier-corrected method was applied, whereas MP-PRESSO raw method was used otherwise.

### Causal associations of mental disorders on frailty

**Fig. 2:**
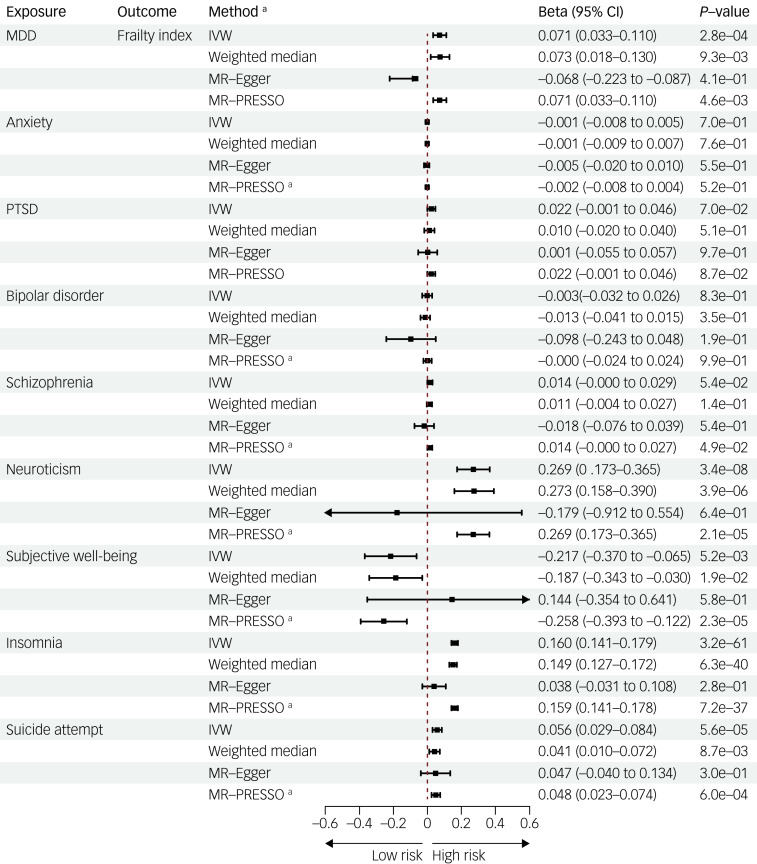
Mendelian randomisation estimates for the causal associations of mental disorders on frailty. IVW, inverse-variance weighted; MDD, major depressive disorder; MR-Egger, Mendelian randomisation Egger; MR-PRESSO, Mendelian randomisation pleiotropy residual sum and outlier; PTSD, post-traumatic stress disorder. ^a^ MP-PRESSO outlier-corrected method was applied, whereas MP-PRESSO raw method was used otherwise.

As depicted in Fig. 2, we conducted reverse Mendelian randomisation analyses to explore the potential causal effects of mental disorders on frailty. Our findings disclosed significant associations between genetic liability to MDD and frailty (*β* = 0.071, 95% CI 0.033–0.110, *P* = 2.8 × 10^−^⁴). Neuroticism exhibited a positive correlation with frailty (*β* = 0.269, 95% CI 0.173–0.365, *P* = 3.4 × 10^−^⁸). Additionally, insomnia (*β* = 0.160, 95% CI 0.141–0.179, *P* = 3.2 × 10^−^⁶¹) and suicide attempt (*β* = 0.056, 95% CI 0.029–0.084, *P* = 3.4 × 10^−^⁵) were associated with an increased risk of frailty. Moreover, subjective well-being exhibited a suggestive negative association with frailty (*β* = −0.217, 95% CI −0.370 to −0.065, *P* = 5.2 × 10^−^³). Consistent findings were identified with the anxiety summary statistics from the FinnGen consortium, as shown in Supplementary Table 3. The weighted median, MR-PRESSO and MVMR methods also yielded consistent results (Fig. 2 and Supplementary Table 5**)**. Similar outcomes were also observed when the obesity- and smoking-related SNPs were excluded (Supplementary Table 4). Notably, no statistically significant causal effects were observed for anxiety, PTSD, bipolar disorder and schizophrenia on frailty.

### Sensitivity analysis

We also conducted a replication analysis with an additional GWAS data-set focusing on frailty assessed by the frailty phenotype. The bidirectional causal relationship between frailty and mental disorders remained consistent, except for the causal effect of subjective well-being on frailty in the replication analysis (Table 3). After the adjustment for potential sample overlap by using the MR-APSS method, the bidirectional causal effects between four mental disorders (neuroticism, subjective well-being, insomnia and suicide attempt) and frailty remained robust (Supplementary Table 6). Additionally, MR-Egger intercept detected horizontal pleiotropy between insomnia and frailty, but this did not affect the robustness of Mendelian randomisation estimation in the present study because we used MVMR analysis with confounding excluded, and replication analysis to validate our results. Additionally, Cochran's *Q*-test detected heterogeneity among some causal relationships between frailty and mental disorders, but this did not affect the overall robustness of our Mendelian randomisation estimates (Table 4). Our study utilised the IVW analysis under a multiplicative random-effects model, effectively addressing the heterogeneity. Notably, none of the SNPs were removed through Steiger filtering, indicating the correct orientation of the inferred relationships. Furthermore, the leave-one-out analysis did not identify individual SNPs that could bias the IVW results (Supplementary Fig. 1). The symmetry of the funnel plots further underscored the reliability of our findings (Supplementary Fig. 2).

**Table 3:** Mendelian randomisation results for the replication analyses using the frailty data assessed by frailty phenotype

Exposure	Outcome	Method	*β* (95% CI)^a^	Odds ratio (95% CI)^b^	*P*-value	*P*-value for Cochran's *Q*/MR-Egger intercept
Frailty phenotype	MDD	IVW	−	2.54 (1.59–4.08)	1.0 × 10^−4^	1.2 × 10^−6^
		Weighted median	−	1.92 (1.16–3.16)	0.011	
		MR-Egger	−	2.02 (0.29–13.95)	0.48	0.81
		MR-PRESSO ^c^	−	2.44 (1.60–3.72)	3.3 × 10^−5^	
Frailty phenotype	Anxiety	IVW	−	3.94 (2.04–7.63)	4.7 × 10^−5^	0.98
		Weighted median	−	4.68 (1.34–16.40)	0.017	
		MR-Egger	−	5.91 (0.12–283.62)	0.38	0.83
		MR-PRESSO	−	3.94 (2.04–7.63)	3.2 × 10^−4^	
Frailty phenotype	PTSD	IVW	−	3.06 (1.75–5.36)	9.1 × 10^−5^	0.063
		Weighted median	−	3.55 (1.76–7.14)	4.0 × 10^−4^	
		MR-Egger	−	4.58 (0.50–41.59)	0.19	0.71
		MR-PRESSO	−	3.06 (1.75–5.36)	5.0 × 10^−4^	
Frailty phenotype	Neuroticism	IVW	0.21 (0.02–0.40)	−	0.034	9.0 × 10^−8^
		Weighted median	0.21 (0.02–0.40	−	0.027	
		MR-Egger	0.00 (−0.73 to 0.74)	−	0.37	
		MR-PRESSO ^c^	0.17 (0.01–0.33)	−	0.046	
Frailty phenotype	Insomnia	IVW	0.70 (0.41–0.99)	−	2.5 × 10^−6^	1.2 × 10^−5^
		Weighted median	0.65 (0.37–0.94)	−	6.6 × 10^−6^	
		MR-Egger	1.00 (−0.09 to 2.09)	−	0.085	0.58
		MR-PRESSO ^c^	0.71 (0.47–0.95)	−	8.5 × 10^−6^	
Frailty phenotype	Suicide attempt	IVW	−	1.79 (0.99–3.25)	0.056	5.4 × 10^−6^
		Weighted median	−	1.78 (1.00–3.14)	0.49	
		MR-Egger	−	4.03 (0.43–37.85)	0.24	0.47
		MR-PRESSO	−	1.40 (0.85–2.32)	0.20	
MDD	Frailty phenotype	IVW	0.05 (0.01–0.09)	−	0.0088	0.040
		Weighted median	0.07 (0.03–0.11)	−	6.1 × 10^−4^	
		MR-Egger	−0.06 (−0.20 to 0.09)	−	0.47	0.18
		MR-PRESSO	0.05 (0.01–0.09)	−	0.034	
Neuroticism	Frailty phenotype	IVW	0.14 (0.06–0.22)	−	3.9 × 10^−4^	1.8 × 10^−6^
		Weighted median	0.10 (0.02–0.17)	−	0.010	
		MR-Egger	−0.08 (−0.70 to 0.54)	−	0.81	
		MR-PRESSO	0.15 (0.09–0.20)	−	1.0 × 10^−4^	
Subjective well-being	Frailty phenotype	IVW	−0.05 (−0.12 to 0.03)	−	0.24	0.032
		Weighted median	−0.05 (−0.13 to 0.03)	−	0.24	
		MR-Egger	0.21 (−0.02 to 0.43)	−	0.094	0.038
		MR-PRESSO ^c^	−0.02 (−0.08 to 0.04)	−	0.53	
Insomnia	Frailty phenotype	IVW	0.08 (0.06–0.09)	−	1.7 × 10^−25^	4.0 × 10^−32^
		Weighted median	0.08 (0.07–0.09)	−	4.7 × 10^−28^	
		MR-Egger	0.02 (−0.03 to 0.07)	−	0.48	0.033
		MR-PRESSO	0.02 (0.07–0.09)	−	4.4 × 10^−23^	
Suicide attempt	Frailty phenotype	IVW	0.04 (0.03–0.06)	−	8.4 × 10^−8^	0.0039
		Weighted median	0.03 (0.02–0.05)	−	4.5 × 10^−4^	
		MR-Egger	0.01 (−0.04 to 0.06)	−	0.71	0.18
		MR-PRESSO	0.04 (0.03–0.06)	−	5.0 × 10^−6^	

MR-Egger, Mendelian randomisation Egger; MDD, major depressive disorder; IVW, inverse-variance weighted; MR-PRESSO, Mendelian randomisation pleiotropy residual sum and outlier; PTSD, post-traumatic stress disorder.

a. *β* is presented for the analyses of continuous/ordinal outcomes.

b. OR is presented for the analyses of binary/dichotomous outcomes.

c. MP-PRESSO outlier-corrected method was applied, whereas MP-PRESSO raw method was used otherwise.

**Table 4:** Sensitivity analysis of the bidirectional causal association between frailty and mental disorders

Exposure	Outcome	Cochran's *Q*-statistic		MR-Egger intercept tests	
		*Q*-value	*P-*value	Intercept	*P-*value
Frailty index	MDD	17.33	0.14	0.013	0.71
Frailty index	Anxiety	6.26	0.90	0.041	0.64
Frailty index	PTSD	15.91	0.25	0.019	0.38
Frailty index	Bipolar disorder	51.83	1.4 × 10^−6^	0.017	0.55
Frailty index	Schizophrenia	58.17	2.0 × 10^−8^	−0.053	0.068
Frailty index	Neuroticism	23.07	0.027	0.015	0.30
Frailty index	Subjective well-being	13.60	0.018	−0.046	0.10
Frailty index	Insomnia	50.12	2.8 × 10^−6^	0.024	0.055
Frailty index	Suicide attempt	24.51	0.017	−0.056	0.21
MDD	Frailty index	8.81	0.55	0.008	0.10
Anxiety	Frailty index	120.26	1.8 × 10^−4^	6.9 × 10^−4^	0.63
PTSD	Frailty index	25.89	0.10	0.0027	0.43
Bipolar disorder	Frailty index	132.10	5.4 × 10^−11^	0.0064	0.20
Schizophrenia	Frailty index	375.39	3.9 × 10^−22^	0.0022	0.25
Neuroticism	Frailty index	35.21	0.019	0.0095	0.24
Subjective well-being	Frailty index	33.94	0.0035	−0.0080	0.16
Insomnia	Frailty index	289.19	3.5 × 10^−11^	0.0055	4.9 × 10^−4^
Suicide attempt	Frailty index	72.28	0.0013	6.2 × 10^-4^	0.83

MR-Egger, Mendelian randomisation Egger; MDD, major depressive disorder; PTSD, post-traumatic stress disorder.

## Discussion

In the present study, we performed a bidirectional Mendelian randomisation analysis to evaluate the associations between frailty and mental disorders. Our results demonstrated that genetically predicted frailty was linked to an elevated risk of MDD, anxiety, PTSD, neuroticism and insomnia. Additionally, the reverse Mendelian randomisation analysis provided evidence of a causal association between frailty and a genetic predisposition to MDD, neuroticism, insomnia and suicide attempt. Furthermore, our results demonstrated a suggestive causal association between frailty and suicide attempt, as well as subjective well-being and frailty. This Mendelian randomisation analysis offers a more comprehensive understanding of the causal associations between frailty and nine mental disorders.

A growing body of epidemiological studies have revealed bidirectional associations between frailty and mental disorders. However, the evidence has primarily been derived from cross-sectional, longitudinal and case–control studies. Most previous studies have focused on exploring only one direction of this association, whether from frailty to mental disorders or *vice versa*, with very few simultaneously investigating the bidirectional relationship, especially within a population-based prospective design. A meta-analysis of 24 studies has established that individuals living with frailty face an elevated risk of depression.^24^ Furthermore, a cross-sectional study revealed that both pre-frail and frail elderly individuals exhibited a heightened risk of depression compared with the general elderly population.^25^ In a population-based cohort of 12 844 individuals aged 65 years and older, it was observed that depression was associated with a 59% higher risk of developing frailty.^26^ In another cross-sectional observational study, insomnia was identified as an independent risk factor for frailty, even after adjusting for sociodemographic characteristics and comorbidity.^27^ Tang et al found that sleep-onset insomnia was linked to an elevated risk of frailty in older individuals.^28^ Also, a recent meta-analysis of 12 observational studies involving 16 895 people demonstrated that insomnia was independently associated with increased risk of frailty in the older population.^29^ Liu et al also identified difficulty initiating sleep and depressive symptoms as independent risk factors for frailty among community-dwelling older adults in the USA.^30^ Cumulative evidence suggests that efforts to reduce neuroticism may play a role in delaying the onset of frailty.^31^ Our Mendelian randomisation results also reinforced a robust bidirectional causal relationship between frailty and MDD, neuroticism and insomnia. Importantly, this was confirmed with two distinct frailty data-sets, including frailty index and frailty phenotype GWAS data-sets.

A systematic review of 20 cross-sectional studies and one longitudinal study found that geriatric individuals with frailty are more likely to experience anxiety compared with their robust counterparts.^32^ A 5-year follow-up of the Lifelines cohort study further reported that frailty is associated with the onset and persistence of anxiety disorders in both younger and older adults.^33^ Additionally, a cross-sectional study found that individuals with mild or severe frailty have a higher risk of PTSD. Our Mendelian randomisation data also revealed a unidirectional causal effect of frailty on anxiety and PTSD. The findings suggest that individuals living with frailty should undergo assessments for anxiety and PTSD, and receive tailored support.

Frailty has also been documented to increase the risk of suicide attempt in prior investigations.^5,34^ A cross-sectional study revealed that individuals with cognitive frailty had a heightened susceptibility to experiencing suicidal ideation.^34^ In a study involving veterans aged 65 years and older, frailty was linked to an elevated risk of suicide attempts, and lower levels of frailty were associated with a greater risk of suicide deaths.^5^ Furthermore, low levels of psychological well-being could exacerbate the frailty status, contributing to the worse physical health and lower life quality of older individuals with frailty.^35^ The current Mendelian randomisation study indicated suggestive relationships between frailty and suicide attempt (*P* = 0.027), as well as subjective well-being and frailty (*P* = 0.0052). However, these associations did not reach statistical significance after adjusting for multiple testing (*P* > 0.0018). Therefore, future GWAS with larger sample sizes are warranted to investigate the causal relationships between frailty and suicide attempt, as well as subjective well-being and frailty, which is of utmost importance for advancing the treatment of frailty and mental disorders.

No bidirectional association between frailty and bipolar disorder or schizophrenia was observed in our Mendelian randomisation study. Although a prospective study demonstrated that frailty prevalence is significantly higher among patients with bipolar disorder,^6^ and previous observational studies showed a positive association between frailty and schizophrenia symptoms,^9^ the null associations observed in our analyses are likely reliable, as we used two complementary measures of frailty in the Mendelian randomisation analysis. Our findings suggest that earlier observational studies may have been influenced by biases, and a direct relationship between frailty and bipolar disorder or schizophrenia may not exist. Several factors, such as differences in frailty assessment, gender, demographic characteristics and other confounding variables, could potentially explain the discrepancies. Additionally, it is possible that population heterogeneity contributed to the absence of a causal relationship, and further studies using GWAS data with larger and more representative samples are needed to confirm our findings.

The underlying mechanism of the bidirectional relationship between frailty and mental disorders remains elusive. Several hypotheses may shed light on this complex causal association. First, unhealthy lifestyle factors such as low physical activity, imbalanced diet, smoking and alcohol consumption, and comorbidities like falls, cognitive impairment and cardiometabolic diseases, may contribute to this bidirectional connection.^10,36^ Additionally, accelerated biological ageing may provide support for this two-way relationship. Growing evidence has demonstrated that shortened leukocyte telomere length is associated increased risk of mental disorders, as well as frailty.^37^ Furthermore, shared risk factors and pathophysiological pathways, including chronic inflammation, oxidative stress, mitochondrial dysfunction and stress hormones, may act as common biological pathways in both directions.^38^ For instance, it has been demonstrated that genetic downregulation of interleukin-6 signalling is linked to a reduced risk of frailty.^39^ Similarly, higher serum levels of interleukin-6 and interleukin-10 have been linked to a greater risk of mental disorders.^40^ Nevertheless, the aetiological model governing the intricate bidirectional causal relationships between frailty and mental disorders necessitates further research to delve into the specific mechanisms underpinning this complex association.

The findings of this bidirectional Mendelian randomisation study have important implications for both public health and clinical practice. The identified bidirectional causal associations underscore the need for prioritising early frailty screening in individuals with depression, neuroticism, and insomnia, as well as the timely provision of psychological support for frail individuals. These measures may help reduce adverse outcomes, including disability and diminished quality of life. Furthermore, it is notable that both mental disorders and frailty share modifiable risk factors, such as smoking and physical inactivity. Implementing interventions to prevent or treat one condition may offer protection against the development or progression of the other.

A major strength of the present study was the bidirectional Mendelian randomisation study design, which effectively minimised the influence of confounding variables, the potential for reverse causality and nondifferential exposures. Additionally, secondary analyses, including MR-Egger, weighted median and MR-PRESSO, were conducted to fortify result consistency and robustness, and implementation of MVMR with adjustment for confounding factors further solidified the reliability of inferring a bidirectional causal association between frailty and mental disorders. Finally, two GWAS data-sets, assessing frailty through the frailty index and frailty phenotype, were employed. Consistently matching results across these two data-sets provided additional affirmation of our findings. Nonetheless, our study does bear certain limitations. First, as this Mendelian randomisation study was conducted exclusively using GWAS summary statistics of individuals of European descent, whether the bidirectional causal association between frailty and mental disorders extends to other ethnicities warrants further investigation. Second, although we conducted multiple sensitivity analyses to identify and address the issue of horizontal pleiotropy, it is a significant concern in the context of Mendelian randomisation. It is important to acknowledge that complete elimination of bias owing to horizontal pleiotropy remains challenging, as these pleiotropic effects may manifest extensively across the genome. Third, it is also important to note that assessments of mental disorders were included in the frailty index. Because of the use of summary data, we were unable to perform a sensitivity analysis that excluded variables related to mental disorders. However, we believe this limitation would not significantly affect our results. The GWAS of the frailty index included only a small proportion (approximately 2%) of participants who reported depression and/or anxiety. Additionally, items directly related to mental disorders constituted only a minor part of the frailty index, with seven out of 49 items in the UK Biobank and three out of 44 items in TwinGene being related to mental health. Regrettably, we were unable to perform additional stratified analyses or investigate non-linear correlations between frailty and mental disorders, primarily because our study relied on publicly available summary-level data.

In summary, our study provides significant evidence of a causal link between frailty and an increased risk of MDD, anxiety, PTSD, neuroticism and insomnia. Bidirectionally, our research findings have also reinforced the detrimental impact of MDD, neuroticism, insomnia and suicide attempts on frailty. Moreover, our results have hinted at the suggestive causal relationship between frailty and suicide attempts, as well as subjective well-being and frailty. These discoveries hold significant promise for informing the development of intervention strategies aimed at mitigating the substantial burden posed by mental disorders and frailty.

## Supporting information

Zhou et al. supplementary material 1 Zhou et al. supplementary material

Zhou et al. supplementary material 2 Zhou et al. supplementary material

Zhou et al. supplementary material 3 Zhou et al. supplementary material

## Data Availability

Publicly available data-sets were utilised in Mendelian randomisation analysis. All relevant data for the study are either included in the article or provided as supplementary information.
