# Supplementary material for: Casual associations between frailty and nine mental disorders: bidirectional Mendelian randomisation study
Source: BJPsych Open. 2025 Feb 3;11(2):e28. doi: 10.1192/bjo.2024.835 (PMC11822947; doi:10.1192/bjo.2024.835)
Supplement: Zhou et al. supplementary material 2 — Zhou et al. supplementary material [file S2056472424008354sup002.docx]

Supplementary file

Association between frailty and mental disorders: a bidirectional Mendelian randomization study


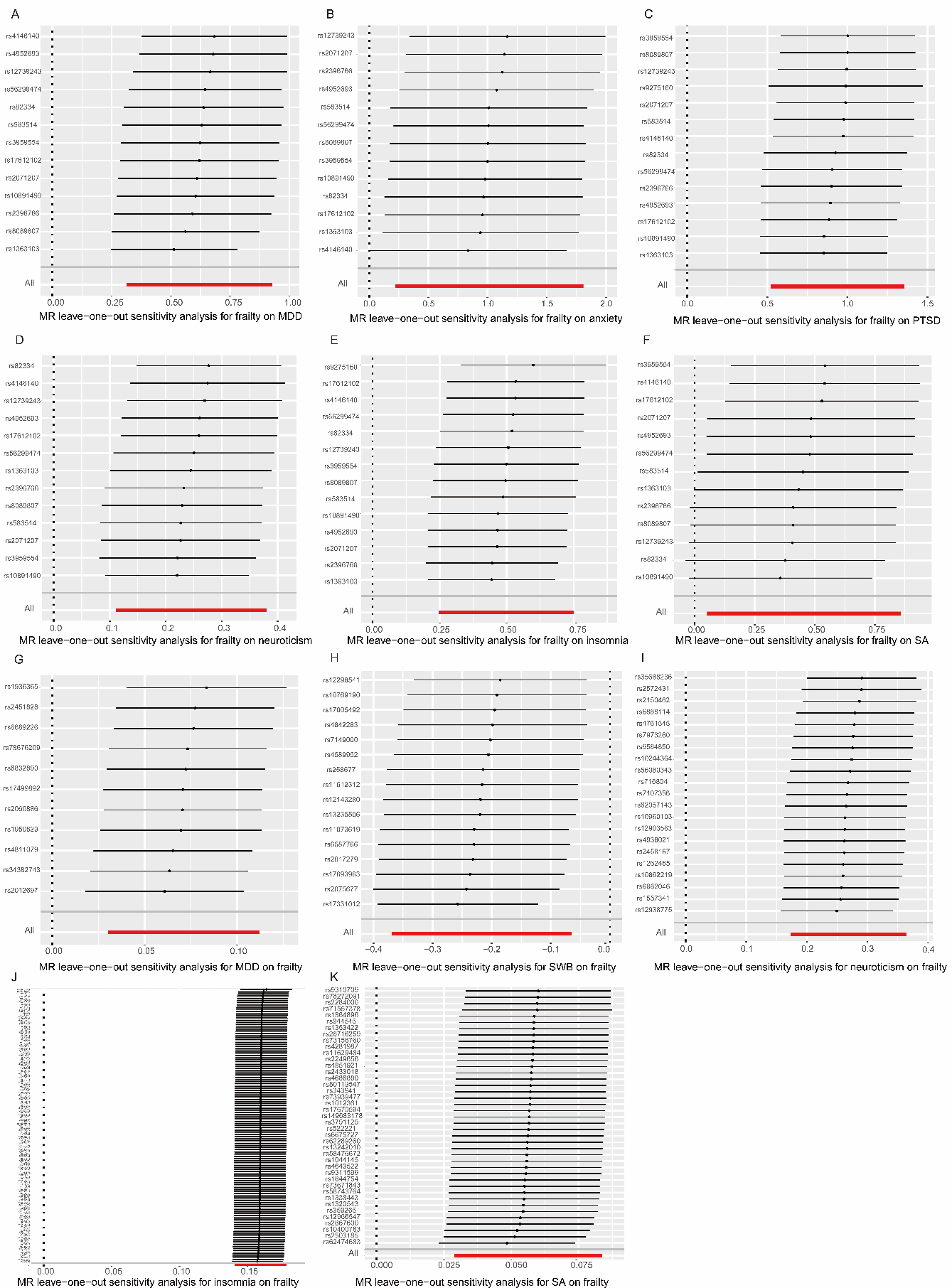


**Supplementary Figure S1. The leave-one-out analysis plots of Mendelian randomization analysis.** (A) Frailty on MDD; (B) Frailty on anxiety; (C) frailty on PTSD; (D) frailty on neuroticism; (E) Frailty on insomnia; (F) Frailty on SA; (G) MDD on frailty; (H) Neuroticism on frailty; (I) SWB on frailty; (J) Insomnia on frailty; (K) SA on frailty. MDD, major depressive disorder; PTSD, post-traumatic stress; SWB, subjective well-being.


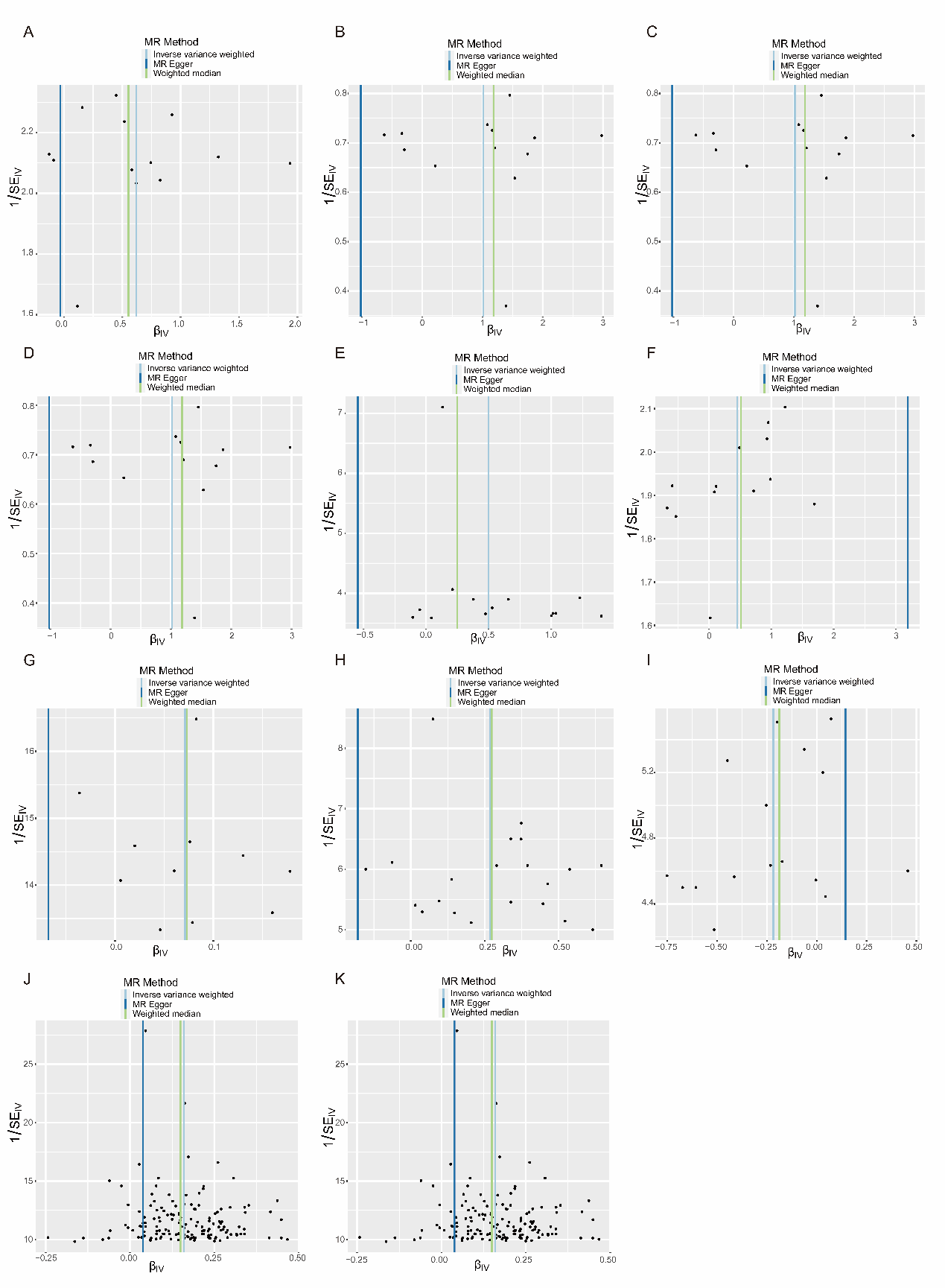


**Supplementary Figure S2. The Funnel plots of Mendelian randomization analysis.** (A) Frailty on MDD; (B) Frailty on anxiety; (C) frailty on PTSD; (D) frailty on neuroticism; (E) Frailty on insomnia; (F) Frailty on SA; (G) MDD on frailty; (H) Neuroticism on frailty; (I) SWB on frailty; (J) Insomnia on frailty; (K) SA on frailty. MDD, major depressive disorder; PTSD, post-traumatic stress; SWB, subjective well-being.
